# Does chemotherapy or radiotherapy affect the postoperative complication in breast cancer patients who underwent immediate breast reconstruction with tissue expander?

**DOI:** 10.1186/s12885-020-07729-w

**Published:** 2021-01-22

**Authors:** Sung Mi Jung, Byung-Joon Jeon, Jinsun Woo, Jai Min Ryu, Se Kyung Lee, Byung-Joo Chae, Jonghan Yu, Seok Won Kim, Seok Jin Nam, Jai-Kyong Pyon, Goo-Hyun Mun, Sa Ik Bang, Jeong Eon Lee

**Affiliations:** 1 Division of Breast Surgery, Department of Surgery, Samsung Medical Center, Sungkyunkwan University School of Medicine, Irwon-ro 81, Gangnam-gu, 06351 Seoul, South Korea; 2 Department of Plastic Surgery, Samsung Medical Center, Sungkyunkwan University School of Medicine, Irwon-ro 81, Gangnam-gu, 06351 Seoul, South Korea

**Keywords:** Breast neoplasm, Chemotherapy, Adjuvant, Postoperative complications, Radiotherapy, Tissue expansion devices

## Abstract

**Background:**

Immediate breast reconstruction with tissue expander in breast cancer patients who were expected to receive adjuvant therapy, such as chemotherapy or radiotherapy, has been a topic of debate. Postoperative complications from tissue expander procedures can delay the timing of adjuvant treatment and subsequently increase the probability of recurrence. The purpose of this study was to identify the impact of chemotherapy and radiotherapy on postoperative complications in patients who underwent immediate reconstruction (IR) using tissue expander.

**Methods:**

We conducted a retrospective study of 1081 breast cancer patients who underwent mastectomy and IR using tissue expander insertion between 2012 and 2017 in Samsung Medical Center. The patients were divided into two groups based on complications (complication group vs. no complication group). Complication group was regarded to have surgical removal or conservative treatment based on clinical findings such as infection, capsular contracture, seroma, hematoma, rupture, malposition, tissue viability, or cosmetic problem. The complication group had 59 patients (5.5%) and the no complication group had 1022 patients (94.5%).

**Results:**

In univariate analysis, adjuvant radiotherapy and adjuvant chemotherapy were significantly associated with postoperative complications. In multivariate analysis, however, only higher pathologic N stage was significantly associated with postoperative complications (*p* < 0.001). Chemotherapy (*p* = 0.775) or radiotherapy (*p* = 0.825) were not risk factors for postoperative complications.

**Conclusions:**

IR with tissue expander after mastectomy may be a treatment option even when the patients are expected to receive adjuvant chemotherapy or radiotherapy. These results will aid patients who are concerned about the complications of IR caused by chemotherapy or radiotherapy determine whether or not to have IR.

**Trial registration:**

Patients were selected and registered retrospectively, and medical records were evaluated.

## Background

Breast cancer is the second most common cancer among women in the United States. In the United States, 250,520 new cases of female breast cancer were reported, and 42,000 women died of this cancer in 2017. For every 100,000 women, 125 new female breast cancer cases were reported and 20 women died of this cancer. The global burden of breast cancer in women, measured by incidence or mortality, is substantial and rising in several countries [1, 2].

Mastectomy with immediate reconstruction (IR) is one of the treatment option for breast cancer. Breast reconstruction after mastectomy has a positive impact on patients’ psychosocial and sexual well-being. Moreover, immediate breast reconstruction can provide patients with the opportunity to reduce one additional surgery and costs instead of undergoing two separate procedures [3–7].

The performance of mastectomy followed by IR with a tissue expander in patients who were expected to receive adjuvant chemotherapy or radiotherapy has been debated. It is important to identify how adjuvant chemotherapy or radiotherapy affects surgical site complications in patients who have undergone IR. Postoperative complications related to tissue expander can miss the timing of adjuvant chemotherapy or radiotherapy and subsequently increase the probability of recurrence. Some previous studies reported that the incidence of postoperative complications caused by chemotherapy or radiotherapy in patients with tissue expander was higher than in patients without tissue expander [8, 9]. Other studies have reported that immediate breast reconstruction may be a feasible surgical option in patients who were expected to receive adjuvant chemotherapy or radiotherapy and that it did not significantly affect the timing of adjuvant treatment or postoperative complications [10, 11].

The aim of this study was to determine the impact of chemotherapy and radiotherapy on postoperative complications in patients who received mastectomy and IR using tissue expander insertion.

## Methods

### Patients

**Fig. 1:**
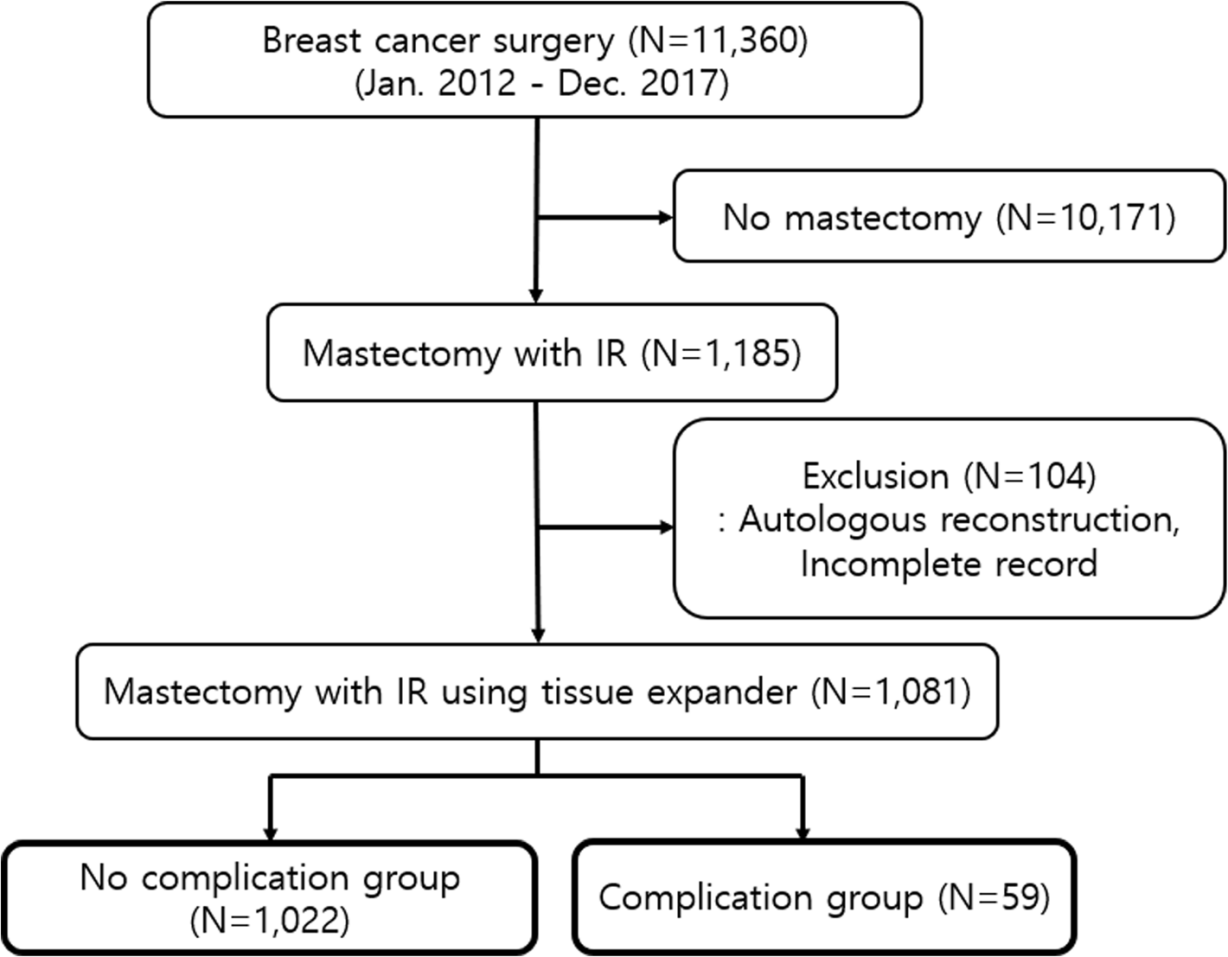
Schematic flow of patients

This study was retrospectively performed on breast cancer patients who underwent mastectomy and IR using tissue expander from January 2012 to December 2017 in Samsung Medical Center. The inclusion criteria included a total of 1081 female breast cancer patients who underwent nipple-sparing mastectomy or skin-sparing mastectomy with IR using tissue expander. The patients who received autologous reconstruction with deep inferior epigastric perforator free flap, latissimus dorsi flap or other tissues were excluded from this study (Fig. 1). There was no risk-reducing mastectomy case in the inclusion criteria. The pathologic breast cancer staging followed the National Comprehensive Cancer Network guidelines version 5.2020 and adjuvant therapy, such as chemotherapy or radiotherapy, was determined by stage and subtype [12].

Inclusion criteria were periodically monitored through physical examination, imaging and laboratory tests that were conducted at intervals of 6 months and 1 year, depending on the stage. The median follow-up was 448 days (range 27–2423 days). The patients were divided based on postoperative complications (complication group vs. no complication group). Complication group was regarded not only to have surgical removal of the tissue expander or implant which inserted after removing the tissue expander, but also to receive conservative treatment without surgical removal. Clinicians decided whether to have surgical removal or conservative treatment based on clinical findings such as infection, capsular contracture, seroma, hematoma, rupture, malposition, tissue viability, or cosmetic problem according to the Clavien-Dindo classification (CDC); CDC show acceptance and reproducibility through presumed parameters of severity of morbidity by correlating the various grades of complications that do not require treatment, or require treatment such as medication or surgery, or lead to death [13]. Surgical site infection (SSI) was diagnosed if there were clinical symptoms such as erythema, discharge, abscess, febrile sense, swelling, or tenderness, with or without isolation of pathogenic bacteria by the judgement of the clinicians. Patients who had signs of infection received surgical removal or conservative treatment with antibiotics. Surgical intervention for SSI was performed if the signs of infection continued despite to empirical antibiotic treatment, or rapid treatment of SSI was required due to planned adjuvant chemotherapy or radiotherapy. Capsular contracture was regarded according to Baker classification system with grade III (moderately contracture with firmness) or grade IV (severe contracture with symptom). Seroma or hematoma was considered as complication if surgical intervention or aspiration was required. Rupture or recurrence was found in breast ultrasound or magnetic resonance imaging performed during follow-up with clinical findings.

The data, including demographic factors, pathologic findings, and perioperative treatment were collected from the electronic medical records after Institutional Review Board Approval in Samsung Medical Center (IRB file no. 2019–10-146). Signed informed consent from the patients was not required.

### Statistical analysis

Statistical analyses were performed using SPSS Statistics versions 25 (IBM Corp., Armonk, NY, USA). The continuous variables were compared using the Student’s t test between the two groups and the results are described as the mean and standard deviation with the range. The Chi-squared test and Fisher’s exact test were conducted to compare the categorical variables between the two groups and the categorical variables are reported as number and percentage. All significant risk factors for postoperative complications were analyzed in univariate and binary logistic regression for multivariate analysis. Confidence intervals (95%) and odds ratios were calculated and statistical significance was defined as *p* < 0.05 in all tests.

## Results

### Characteristics of patients

A total of 11,360 patients underwent surgery for breast cancer from January 2012 to December 2017 in Samsung Medical Center. Of these, 1185 patients who underwent mastectomy with IR and 1081 patients who underwent IR with tissue expander insertion were included. There were 1022 patients (94.5%) without postoperative complications (no complication group) and 59 patients (5.5%) with postoperative complications (complication group) (Fig. 1). The median follow-up was 448 days (range 27–2423 days).

The baseline characteristics of the patients between the two groups were well balanced. The mean age of the patients at the time of breast cancer surgery was 43.29 years in the no complication group and 42.29 years in the complication group. There was no significant difference in the number the patients less than 40 years old and those more than 40 years old between the two groups. The mean body mass index (BMI) was 21.96 kg/m2 in the no complication group and 22.40 kg/m2 in the complication group. There was no significant difference in the number of underweight, normal, or overweight patients defined by World Health Organization criteria (Asia-Pacific region) between the two groups. The incidence of patients with a history of diabetes mellitus (DM) or smoking was not significantly different between the two groups. Prior smokers were defined as those who quit smoking more than 6 months earlier (Table 1).

**Table 1:** Baseline characteristics of patients

Variable	Total, N Mean ± SD	No complication group, N (%) Mean ± SD	Complication group, N (%) Mean ± SD	*p*
Number of Patients	1081	1022 (94.5)	59 (5.5)	
Age (years)	43.29 ± 7.40	43.29 ± 7.35	42.29 ± 8.30	0.998
Age group				0.985
< 40 years	331	313 (94.6)	18 (5.4)	
≥ 40 years	750	709 (94.5)	41 (5.5)	
BMI (kg/ ***m***^**2**^)	21.98 ± 2.93	21.96 ± 2.91	22.40 ± 3.19	0.266
BMI group				0.383
< 18.5	75	73 (97.3)	2 (2.7)	
18.5–22.9	668	633 (94.8)	35 (5.2)	
≥ 23	338	316 (93.5)	22 (6.5)	
DM				0.595
Yes	16	15 (93.8)	1 (6.3)	
No	1065	1007 (94.6)	58 (5.4)	
Smoking				0.439
Never	1048	991 (94.6)	57 (5.4)	
Prior	7	6 (85.7)	1 (14.3)	
Current	26	25 (96.2)	1 (3.8)	

*BMI* Body mass index, *DM* Diabetes mellitus

The pathologic characteristics of lymphovascular invasion (LVI), pathologic N stage, and pathologic prognostic stage were significantly associated with postoperative complications. LVI was detected more often in the complication group than in the no complication group (*p* = 0.001). The proportion of patients with higher than pathologic T1 stage cancer was 91.5% in the complication group and 79.7% in the no complication group. The proportion of patients with higher than pathologic N1 stage cancer was 54.2% in the complication group and 24.9% in the no complication group. There was a significant difference in the proportion of patients who were diagnosed with pathologic prognostic stage III cancer (*p* = 0.017). Invasive cancer was diagnosed 77.8% patients, and there was no significantly difference in two groups. The patients with ER-positive or PR-positive were 77.9%, and with HER2-negative were 61.4% in two groups. The histologic feature and subtype were not significantly different between the two groups (Table 2).

**Table 2:** Clinicopathologic characteristics of patients

Variable	Total, N	No complication group, N (%)	Complication group, N (%)	*p*
Number of Patients	1081	1022 (94.5)	59 (5.5)	
Location				0.032
Right	514	484 (94.2)	30 (5.8)	
Left	499	478 (95.8)	21 (4.2)	
Bilateral	68	60 (88.2)	8 (11.8)	
Histopathology				0.939
Invasive cancer	841	794 (94.4)	47 (5.6)	
DCIS	199	189 (95.0)	10 (5.0)	
Other	41	39 (95.1)	2 (4.9)	
Nuclear grade				0.966
Low	153	144 (94.1)	9 (5.9)	
Intermediate	663	627 (94.6)	36 (5.4)	
High	265	251 (94.7)	14 (5.3)	
LVI				0.001
Yes	285	258 (90.5)	27 (9.5)	
No	796	764 (96.0)	32 (4.0)	
Pathologic T				0.118
Tis & pCR	212	207 (97.6)	5 (2.4)	
T1	514	484 (94.2)	30 (5.8)	
T2	299	280 (93.6)	19 (6.4)	
≥ T3	56	51 (91.1)	5 (8.9)	
Pathologic N				< 0.001
N0	795	768 (96.6)	27 (3.4)	
N1	227	208 (91.6)	19 (8.4)	
N2	42	33 (78.6)	9 (21.4)	
N3	17	13 (76.5)	4 (23.5)	
Pathologic prognostic stage				0.017
0 & NRT	207	199 (96.1)	8 (3.9)	
I	456	431 (94.5)	25 (5.5)	
II	342	326 (95.3)	16 (4.7)	
III	76	66 (86.8)	10 (13.2)	
ER				0.445
Positive	895	844 (94.3)	51 (5.7)	
Negative	186	178 (95.7)	8 (4.3)	
PR				0.192
Positive	842	792 (94.1)	50 (5.9)	
Negative	239	230 (96.2)	9 (3.8)	
C-erbB-2				0.586
Positive	296	280 (94.6)	16 (5.4)	
Negative	664	630 (94.9)	34 (5.1)	
Unknown	121	112 (92.6)	9 (7.4)	
Subtype				0.880
HR (+)	900	849 (94.3)	51 (5.7)	
HR(−) C-erbB-2(+)	121	116 (95.9)	5 (4.1)	
HR(−) C-erbB-2(−)	56	53 (94.6)	3 (5.4)	
Unknown	4	4 (100.0)	0 (0.0)	

*DCIS* Ductal carcinoma in situ, *LVI* Lymphovasvular invasion, *pCR* Pathologic complete response, *NRT* No residual tumor, *HR* Hormonal receptor

There were significant differences in type of axillary surgery and adjuvant treatments. Patients in the complication group significantly more received axillary lymph node dissection and adjuvant post-mastectomy radiotherapy (PMRT) than patients in no complication group (*p* < 0.001) (Table 3).

**Table 3:** Characteristics of surgical and medical treatment

Variable	Total, N	No complication group, N (%)	Complication group, N (%)	*p*
Number of Patients	1081	1022 (94.5)	59 (5.5)	
Axillary operation				< 0.001
SLNB	876	839 (95.8)	37 (4.2)	
ALND	205	183 (89.3)	22 (10.7)	
PMRT				< 0.001
Yes	193	172 (89.1)	21 (10.9)	
No	888	850 (95.7)	38 (4.3)	
Chemotherapy				0.062
No	599	575 (96.0)	24 (4.0)	
NAC	61	57 (93.4)	4 (6.6)	
Adjuvant chemotherapy	421	390 (92.6)	31 (7.4)	

*SLNB* Sentinel lymph node biopsy, *ALND* Axillary lymph node dissection, *PMRT* Post-mastectomy radiotherapy, *NAC* Neoadjuvant chemotherapy

### Risk factors of postoperative complications

Postoperative complications developed in 59 patients. Twenty-three (39.0%) of the 59 patients developed infections, and empirical antibiotics are administered. Seventeen of the 23 patients were treated with surgical removal, and 14 of the 17 patients had SSI which isolated pathogenic bacteria. Six of the 23 patients were treated with antibiotics without the removal of tissue expanders, and 3 of the 6 patients had SSI which isolated pathogenic bacteria. One of 59 patients was diagnosed with brain metastasis and underwent removal of the tissue expander for brain magnetic resonance imaging scans. One of two patients with persistent seroma formation after adjuvant radiotherapy required the removal of the tissue expander. For cosmetic effects, one patient with rippling had re-operation to change her implant and one patient had scar revision (Table 4).

**Table 4:** Type of postoperative complication

Complication type	Total, N	Surgical removal, N	No removal, N
	59	48	11
Recurrence or Metastasis	4	4	0
Infection	23	17	6
Capsular contracture	10	10	0
Rupture	3	3	0
Malposition	6	5	1
Arm motion limitation	1	1	0
Hematoma	1	0	1
Wound dehiscence	3	2	1
Wound necrosis	4	3	1
Skin erythema & Seroma	1	1	0
Cosmetic surgery^a^	2	1	1
Desmoid tumor	1	1	0

^a^Cosmetic surgery: rippling, scar revision

Eleven (18.6%) out of 59 patients recovered after conservative treatment without removal of the tissue expander or implant, and 48 (81.4%) other patients required operation for complications (Table 4). Fifteen (31.3%) out of 48 patients had their tissue expanders removed and 33 (68.8%) other patients had their implants removed, which were inserted after tissue expander removal. The median time from surgery to the initiation of the adjuvant treatment, chemotherapy or radiotherapy, was 30 days, and adjuvant treatment was delayed in six of 35 patients who required adjuvant chemotherapy or radiotherapy because postoperative complications developed. Five of six patients needed removal of their tissue expanders and one of six patients recovered after conservative treatment without removal of their tissue expander. Five of six patients delayed their adjuvant chemotherapy and one of six patients delayed adjuvant radiotherapy. The median time from mastectomy to complications was 620 days (range 27–2423 days).

In univariate analysis, the pathologic results showed that LVI was associated with a higher risk of postoperative complication than that in patients without LVI (HR = 2.499, 95% CI: 1.469 to 4.250, *p* = 0.001). The patients with higher pathologic N stages had more postoperative complications. The patients with pathologic N2 stage had 7.758 times (95% CI: 3.379 to 17.808, *p* < 0.001) the risk of postoperative complications as the patients with pathologic N0 stage, and those with pathologic N3 stage had 8.752 times (95% CI: 2.677 to 28.612, *p* < 0.001) the risk of the pathologic N0 stage patients. In complication group, the mean amount of metastatic and retrieved lymph nodes were 4.27 and 19.00 in patients with higher pathologic N1 stage, respectively. The patients with pathologic prognostic stage III had 3.769 times (95% CI: 1.428 to 9.947, *p* = 0.007) the risk of postoperative complications as the patients with pathologic prognostic stage 0 or no residual tumor (NRT) after neoadjuvant chemotherapy. Although the patients with axillary lymph node dissection (ALND), PMRT or adjuvant chemotherapy were associated with a higher risk of complications in univariate analysis, however, ALND, PMRT, or adjuvant chemotherapy were not significantly different between the two groups in multivariate analysis. In multivariate analysis, only the pathologic N stage was associated with postoperative complications (*p* < 0.001) (Table 5). The regimens of adjuvant chemotherapy were independently analyzed, as a result, doxorubicin, cyclophosphamide, and docetaxel regimen (AC-D) had 3.304 times (95% CI: 1.483 to 7.361, *p* = 0.003) the risk of postoperative complications (Table 6).

**Table 5:** Univariate and multivariate analysis of risk factors for postoperative complication

	Univariate		Multivariate	
	HR (95% CI)	*p*	HR (95% CI)	*p*
Location				
Right (Ref.)		0.039		0.020
Left	0.709 (0.400–1.256)	0.238	0.659 (0.366–1.185)	0.163
Bilateral	2.151 (0.943–4.907)	0.069	2.331 (0.989–5.490)	0.053
Histopathology				
Invasive cancer (Ref.)		0.939		0.921
DCIS	0.894 (0.444–1.801)	0.754	1.188 (0.508–2.780)	0.691
Other	0.866 (0.203–3.698)	0.846	1.079 (0.241–4.842)	0.921
LVI				
Yes	2.499 (1.469–4.250)	0.001	1.709 (0.946–3.085)	0.076
No (Ref.)				
Pathologic N				
N0 (Ref.)		< 0.001		< 0.001
N1	2.598 (1.417–4.766)	0.002	2.203 (1.163–4.172)	0.015
N2	7.758 (3.379–17.808)	< 0.001	6.823 (2.845–16.361)	< 0.001
N3	8.752 (2.677–28.612)	< 0.001	6.331 (1.831–21.886)	0.004
Pathologic prognostic stage				
0 & NRT (Ref.)		0.025		0.179
I	1.443 (0.640–3.255)	0.377	1.232 (0.461–3.288)	0.677
II	1.221 (0.513–2.905)	0.652	0.054 (0.170–1.713)	0.295
III	3.769 (1.428–9.947)	0.007	0.564 (0.145–2.200)	0.410
Axillary op				
SLNB (Ref.)				
ALND	3.144 (1.826–5.414)	< 0.001	1.450 (0.674–3.123)	0.342
PMRT +				
Yes	2.731 (1.564–4.769)	< 0.001	1.101 (0.468–2.589)	0.825
No (Ref.)				
CTx.				
No (Ref.)		0.067		0.775
NAC	1.681 (0.564–5.015)	0.351	0.625 (0.168–2.328)	0.483
Adjuvant CTx.	1.904 (1.101–3.295)	0.021	0.839 (0.384–1.833)	0.660

*DCIS* Ductal carcinoma in situ, *LVI* Lymphovasvular invasion, *NRT* No residual tumor, *SLNB* Sentinel lymph node biopsy, *ALND* Axillary lymph node dissection, *PMRT* Post-mastectomy radiotherapy, *NAC* Neoadjuvant chemotherapy

**Table 6:** Risk factors for postoperative complication by regimens of adjuvant chemotherapy

Regimen	N	Odds ratios (95% CI)	*p*
Patients	421		
AC	126	0.323 (0.111–0.943)	0.039
AC-D	187	3.304 (1.483–7.361)	0.003
AC-P	24	1.147 (0.257–5.122)	0.857
FAC	47	0.526 (0.121–2.278)	0.390
TC	22	0.583 (0.076–4.482)	0.604
TCH	13	0.000 (0.000–0.000)	0.999
f/u loss	2		

## Discussion

In our study, only the pathologic N stage was significantly associated with higher risk of postoperative complications in patients who received mastectomy and IR using tissue expander. Chemotherapy and radiotherapy did not significantly affect postoperative complications. Of the 1081 patients, 483 patients received adjuvant chemotherapy or radiotherapy and only six out of 483 patients delayed their adjuvant treatment because of postoperative complications.

Breast cancer patients have been able to select immediate breast reconstruction more easily without worrying cost because insurance has covered breast reconstruction for breast cancer patients since April 2015 in Korea. Several studies have demonstrated oncologic safety in the patients who underwent IR following mastectomy [14–16]. In addition, the majority of previous studies on the impact of chemotherapy or radiotherapy on postoperative complications after immediate breast reconstruction reported that chemotherapy or radiotherapy did not increase postoperative complications and immediate breast reconstruction did not affect the initiation of adjuvant treatment [5, 8–11, 17–24]. This study also reported that chemotherapy or radiotherapy did not affect postoperative complications in the patients who underwent immediate breast reconstruction with tissue expander.

Several studies reported that age, BMI, smoking, and DM were risk factors of postoperative complications in patients who underwent immediate breast reconstruction with tissue expanders or implants. Old age, increasing BMI, smoking, and DM can affect postoperative complications related to wound healing, leading to the removal of tissue expanders or implants [8, 25–27]. In this study, the mean age and BMI were 43.29 years and 21.98 kg/m2, respectively, indicating that this study was performed mostly on young, normal-weight patients. Because our study had small numbers of patients with DM or current smokers, the analysis of the impact of DM or smoking on postoperative complications was not powerful enough to determine associations.

Our study showed that the postoperative complication rate was only 5.5% and the median time from mastectomy to complication was 620 days (range 27–2423 days). In addition, it is reported that only higher N stage was associated with postoperative complications. Lymphatic vessels play an important role in wound healing and wound healing is a complex process including inflammation, coagulation, and formation of granulation tissue with angiogenesis and lymphangiogenesis [28]. Metastatic axillary lymph nodes which have architectural distorsion, loss of hilum, or cortical thickness can affect scar formation and sensory nerves in surrounding tissues, therefore, the higher the N stage, the more the removal of this axillary lymph nodes affects the lymph drainage of the arm, which can result in postoperative complication such as breast edema and delayed wound healing [29]. In addition, previous studies have demonstrated that sentinel lymph node biopsy and ALND have association with postoperative complication such as lymphedema, wound infection, and seroma formation [30, 31]. In our study, the most common cause of complications was infections, 2.1%. Some studies have reported that the incidence of seroma formation was 3–85% after breast or axillary surgery and seroma aspiration was a risk factor for SSI [32–35], and in our study, only one of 1081 patients after IR had seroma formation in surgical site and underwent surgical removal of tissue expander. To improve the completion of breast reconstruction, surgeons try to prevent seroma formation at the surgical site by minimizing the dead space and educating patients on how to exercise the arm that is ipsilateral to the breast cancer [34, 36]. Other studies have demonstrated that early drain removal is safe to prevent seroma formation, however, there was no investigation of the timing of drain removal in our study [37, 38].

Although the probability of chemotherapy or radiotherapy increases as the pathologic prognostic stage increases, chemotherapy or radiotherapy was not significantly related to postoperative complication in this study. It has been a controversy whether it is appropriate to do an IR or to do a delayed breast reconstruction in patients with advanced breast cancer. Chemotherapy may be associated toxicity, immunosuppression, and fat necrosis, which may lead to wound healing and PMRT may cause local damage such as fat necrosis, wound dehiscence, flap fibrosis [39, 40]. Therefore, clinicians have not actively recommended IR in patients who were expected to have adjuvant chemotherapy or radiotherapy because it is possible to increase the probability of recurrence by missing the appropriate timing of adjuvant chemotherapy or radiotherapy. However, surgical technique about breast reconstruction with tissue expander has improved over the past years, resulting in more natural, reassuring, and better results. Therefore, breast reconstruction surgery has recently been an indispensable part of breast cancer surgery. In addition to the development of surgical technique, chemotherapy or radiotherapy did not significantly increase postoperative complication and delay timing of adjuvant treatment in our study. In this study, 35 out of 59 patients who had postoperative complications underwent adjuvant chemotherapy or radiotherapy, and only six of 35 patients delayed their adjuvant treatment because of postoperative complication. Five patients suffered from infections during adjuvant chemotherapy and one patient with a headache was diagnosed with brain metastasis during adjuvant radiotherapy. Another 29 of 35 patients who underwent adjuvant treatment developed postoperative complications after adjuvant treatment.

In this study, adjuvant AC-D regimens significantly impact postoperative complications. Specific chemotherapeutic agents may adversely impact wound healing on an immediate tissue expander or implant reconstruction. Changing the chemotherapeutic agents according to side effects may improve postoperative complication and outcomes [8, 41].

This study had some limitations. It was a retrospective review and thus, had selection bias. Also, there are other variables in immediate autologous breast reconstructions because this study was limited to IR with tissue expander. Further studies overcoming these limitations can help to determine the effects of IR.

## Conclusions

Several previous studies have reported oncologic safety and no difference in complications after IR following mastectomy in patients with breast cancer [14–16]. However, many surgeons still hesitate to perform immediate breast reconstruction for patients with high-stage breast cancer. Our data suggested that chemotherapy or radiotherapy were not risk factors for postoperative complications, and did not cause delay adjuvant treatment. This study will help patients who are concerned about the complications of IR caused by chemotherapy or radiotherapy determine whether or not to have IR.

## Data Availability

The datasets used and/or analysed during the current study are available from the corresponding author on reasonable request.

## Abbreviations

ALND: Axillary lymph node dissection
BMI: Body mass index
CDC: Clavien-Dindo classification
DM: Diabetes mellitus
IR: Immediate reconstruction
LVI: Lymphovascular invasion
NRT: No residual tumor
PMRT: Post-mastectomy radiotherapy
SSI: Surgical site infection
